# Carbazolyl-Modified Neutral Ir(III) Complexes for Efficient Detection of Picric Acid in Aqueous Media

**DOI:** 10.3390/s24134074

**Published:** 2024-06-22

**Authors:** Jiangchao Xu, Liyan Zhang, Yusheng Shi, Chun Liu

**Affiliations:** State Key Laboratory of Fine Chemicals, Frontier Science Center for Smart Materials, School of Chemical Engineering, Dalian University of Technology, Linggong Road 2, Dalian 116024, China; xujc@mail.dlut.edu.cn (J.X.); lyzhang@dlut.edu.cn (L.Z.)

**Keywords:** neutral Ir(III) complexes, carbazolyl group, picric acid, AIPE property, photo-induced electron transfer

## Abstract

Based on the electron-deficient property of picric acid (PA), two neutral Ir(III) complexes **1** and **2** modified with the electron-rich carbazolyl groups were synthesized and characterized. Both **1** and **2** exhibit aggregation-induced phosphorescence emission (AIPE) properties in THF/H_2_O. Among them, **2** is extremely sensitive for detecting PA with a limit of detection of 0.15 μM in THF/H_2_O. Furthermore, the selectivity for PA is significantly higher compared to other analytes, enabling the efficient detection of PA in four common water samples. The density functional theory calculations and the spectroscopic results confirm that the sensing mechanism is photo-induced electron transfer (PET).

## 1. Introduction

Since Tang et al. firstly stated the concept of aggregation-induced emission (AIE) in 2001 [1], numerous AIE materials have been reported [2,3,4,5]. The restriction of intramolecular motion (RIM) is the widely accepted AIE mechanism. According to RIM, when aggregates are formed, intramolecular rotation and/or vibration of the corresponding molecules are restricted. This restriction reduces the amount of thermal energy that the molecule consumes as it decays from the electronically excited state to the ground state. Consequently, the energy is released in the form of radiative transitions, which enhances the luminescence of the molecules [6]. Over the past two decades, AIE materials have undergone significant scientific and technological development, leading to a continuously expanding variety of AIE luminogens (AIEgens). The family of AIEgens has grown to include heteroatom compounds, pure hydrocarbons, macromolecules, as well as metallic–organic compounds. Among them, metal–organic complexes with AIE properties have been widely used in various fields, including organic light-emitting diodes [7], bio-imaging [8], disease treatment [9], and explosives detection [10,11].

Designing the structures of ligands to modulate the properties of metal complexes is a useful method in the development of novel functional materials. Carbazolyl, as a kind of electron-rich moiety with excellent hole-transporting ability and a large conjugation plane, is common for modulating molecular structures [12,13,14]. The carbazolyl group could enhance the interaction between Ir(III) complexes and electron-deficient nitroaromatic explosives. For example, Su and co-workers reported two Ir(III) complexes ((PFBHC)_2_Ir(acac) and FIrPicOMPBHCz) containing carbazolyl groups for detecting nitroaromatic explosives in CH_2_Cl_2_, and the limits of detection (LODs) for PA were 29 μM and 10 μM, respectively. The authors suggested that the introduction of the electron-rich carbazolyl groups facilitates the excited-state electron-transfer process [15,16]. Furthermore, Ir(III) complexes featuring carbazolyl groups exhibited significantly enhanced solubility and photostability. In 2023, Di et al. reported two carbazolyl-modified Ir(III) complexes IrCzPh and IrPhCz. Compared to Ir(ppy)_2_(acac), the photostability of both complexes was significantly improved and their AIE properties were activated in MeCN/H_2_O [17]. Moreover, it is evident that the introduction of the carbazolyl group into organic molecules is significant in the detection of explosives. In 2016 and 2017, Verbitskiy and co-workers reported a series of D-π-A type dyes for detecting nitroaromatic explosives such as 2,4,6-trinitrotoluene (TNT), 2,4-dinitrophenol (DNT), and PA. The introduction of the carbazolyl moieties enhances the electron-donating ability of the compounds, enabling the regulation of their absorption and emission properties through the intramolecular charge-transfer (ICT) mechanism [18,19].

Due to its excellent explosive property, 2,4,6-Trinitrophenol, often referred to as picric acid (PA), has been utilized in paints, dyes, pharmaceuticals, and aerospace applications [20,21,22]. However, PA is highly toxic and non-biodegradable, and can lead to liver and kidney dysfunction, conjunctivitis, skin allergic ulcers, and other serious diseases, if it accumulates in the body for a prolonged period [23,24,25,26]. The leakage and misuse of PA could result in the serious contamination of soil and water. As a result, it is critical to develop simple, highly sensitive and selective methods to detect PA, especially in aqueous media. Scientists widely prefer photoluminescence spectroscopy for its exceptional sensitivity, cost-effectiveness, ease of operation, and practical applicability. Therefore, photoluminescence for the detection of PA has attracted much attention.

**Figure 1:**
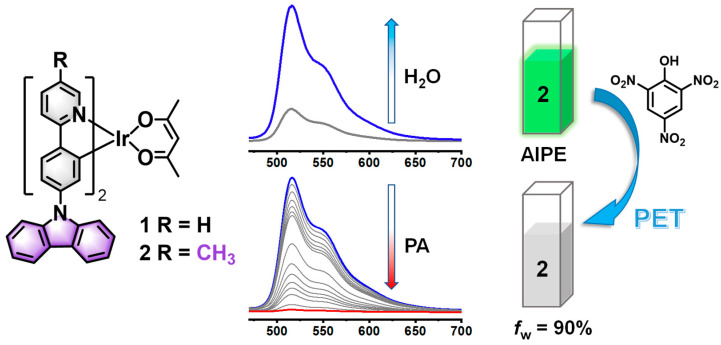
The structures of **1** and **2** and luminescent property of **2** in in THF/H_2_O, and the schematic diagram for the detection of PA.

Our group possesses an enduring interest in investigating the relationship between the structural composition and functional properties of cyclometalating Pt(II) and Ir(III) complexes [27,28,29,30,31]. Recently, we have successfully applied cationic Ir(III) complexes with AIPE properties for the detection of PA in aqueous media with high sensitivity [32,33]. However, the performances of AIPE-active neutral Ir(III) complexes for detecting PA still need to be further explored. In this work, we synthesized two carbazolyl-modified neutral Ir(III) complexes with 2-phenylpyridine derivatives as cyclometalating ligands and acetylacetone as the auxiliary ligand. The corresponding structures of the complexes are shown in Figure 1. The luminescent properties and performance of **2** in the detection of PA in the aqueous media have been investigated in detail.

## 2. Experimental Section

### 2.1. Reagents and Instruments

All raw materials from commercial suppliers were utilized without additional purification. The ^1^H NMR and ^13^C NMR spectra were acquired utilizing a 400 MHz Varian Unity Inova spectrophotometer (Palo Alto, CA, USA). Mass spectra were obtained employing a Bruker Ultraflexetreme MALDI TOF mass spectrometer (Wurzbach, Germany). Photoluminescence quantum yields (*Φ*_PL_) were measured using [Ir(ppy)_2_(acac)] as a standard (*Φ*_PL_ = 0.34 in CH_2_Cl_2_, under deoxygenated conditions). Phosphorescence lifetimes were measured by employing an Edinburgh FLS920 spectrometer (Livingston, Scotland, UK) in a degassed CH_2_Cl_2_ solution and FLS1000 photoluminescence spectrometer (Livingston, Scotland, UK) in unoxygenated THF/H_2_O. UV−Vis absorption spectra were acquired by utilizing an Agilent Cary 100 UV−Vis spectrophotometer (Santa Clara, CA, USA). Emission spectra were recorded utilizing a Hitachi F-7100 fluorescence spectrophotometer (Beijing, China), with luminescence characteristics of **1** and **2** observed under identical instrumental parameters. Density functional theory (DFT) calculations were measured using the B3LYP floods. The 6-31G basis sets were applied for C, H, and O atoms, while the LanL2DZ basis set was utilized for iridium atoms. All computations were executed using Gaussian 16. The dynamic light scattering (DLS) was measured on Malvern ZS90 (Malvern, UK).

### 2.2. Synthesis and Characterizations of ***1*** and ***2***

The Ir(III) complexes were synthesized according to a two-step method reported in the literature [27]. Firstly, IrCl_3_·3H_2_O (0.20 mmol, 70.52 mg) and the cyclometalating ligands (0.50 mmol) were added to 12 mL of anhydrous EtOC_2_H_4_OH/H_2_O (3:1, *V*/*V*). The mixture was stirred for 24 h at 110 °C under N_2_ to obtain a dichloro-bridged intermediate. The dichloro-bridge intermediate without further purification, K_2_CO_3_ (1.00 mmol, 138.21 mg), and acetylacetone (1.00 mmol, 103 μL) were reacted in 12 mL of anhydrous EtOC_2_H_4_OH under stirring and refluxing at 120 °C in N_2_ for 24 h. After reaction, the synthesized complexes were subjected to purification via silica gel column chromatography, employing a solvent system consisting of *n*-hexane and CH_2_Cl_2_ in a 1:1 ratio as the eluting agent, resulting in the complexes **1** and **2**.

### 2.3. Sample Preparation and Sensing of PA

WARNING! The nitroaromatic compounds used in emission spectroscopic studies are highly explosive and must be used safely in small quantities.

Stock solutions of **1** and **2** (100 μM) in THF were prepared firstly. For each suspension of the complexes in THF/H_2_O, 300 μL of the stock solution was mixed with an appropriate volume of THF and deionized water to formulate 3 mL (10 μM) suspensions with various water fractions. The emission spectra and UV−Vis absorption spectra of the samples were each documented. A suspension of **2** (10 μM) with a 90% water fraction was prepared in a 200 mL volumetric flask. Then, 3 mL of each suspension was withdrawn and added into a quartz cuvette, and the emission spectra for each of the 11 blank suspensions were recorded for the calculation of the standard deviation (Figure S8). The solutions of PA were prepared in THF/H_2_O (*f*_w_ = 90%) at concentrations ranging from 0.1 to 50 mM. PA solutions (30 μL) with various concentrations were introduced into cuvettes containing 3 mL of the complex suspension, and the emission spectra were recorded. For selectivity experiments on PA detection, various analytes (30 μL, 30 mM) were added, including nitrobenzene (NB), 1,3-dinitrobenzene (1,3-DNB), nitromethane (NM), *p*-cresol, *m*-cresol, phenol, 4-methoxyphenol (MEHQ), 2-nitrophenol (*o*-NP), and 4-nitrophenol (*p*-NP). Ion interference experiments were conducted to assess the effects of various ionic compounds (30 μL, 30 mM each of CaCl_2_, MnCl_2_, FeCl_2_, NiCl_2_, ZnCl_2_, CoCO_3_, NaHCO_3_, MgSO_4_, CuSO_4_, KF, KBr, and CH_3_COONa) on the suspensions of **2**, where 3 mL of the suspension was used. In competition experiments, PA solutions were added to suspensions of **2** containing the ionic compounds and other analytes, respectively. To assess the real application capacity of **2**, various water samples (tap water from the State Key Laboratory of Fine Chemicals, river water from the Malan River in Dalian, rainwater from Dalian University of Technology, and seawater from Qixianling in Dalian) were used instead of deionized water to formulate suspensions of **2**. Subsequently, 30 μL of 30 mM PA solutions was added to the suspensions, respectively, and the corresponding emission spectra were recorded.

## 3. Results and Discussion

### 3.1. Photophysical and AIPE Properties

The UV−Vis absorption spectra and normalized emission spectra at room temperature of complexes **1** and **2** in THF solution are shown in Figure S3. The photophysical data of both complexes can be found in Table S1. Similar to most Ir(III) complexes, both **1** and **2** exhibit strong absorption bands below 400 nm, which belong to the typical ligand-centered (^1^LC(^1^π-π*)) transitions. The lower energy absorption between 400 and 500 nm is attributed to a combination of metal-to-ligand charge transitions (^1^MLCT/^3^MLCT), ligand-to-ligand charge transitions (^1^LLCT/^3^LLCT), and ligand-centered (^3^LC(^3^π-π*)) leaps. The normalized emission spectra indicate that the maximum emission wavelength and peak shape of **2** remain largely unchanged compared to those of **1**. The phosphorescence lifetimes of **1** and **2** are 1.92 μs and 1.77 μs in degassed CH_2_Cl_2_ at room temperature, respectively (Figure S4), which is typical of phosphorescence emission. The phosphorescence quantum yields of **1** and **2** were measured in degassed CH_2_Cl_2_ using Ir(ppy)_2_(acac) (0.34) as a standard [34]. The results indicate phosphorescence quantum yields of 0.18 for **1** and 0.27 for **2**, respectively. Incorporating an electron-donating group (5-CH_3_) onto the pyridine ring of complex **2** resulted in an increased phosphorescence quantum yield.

**Figure 2:**
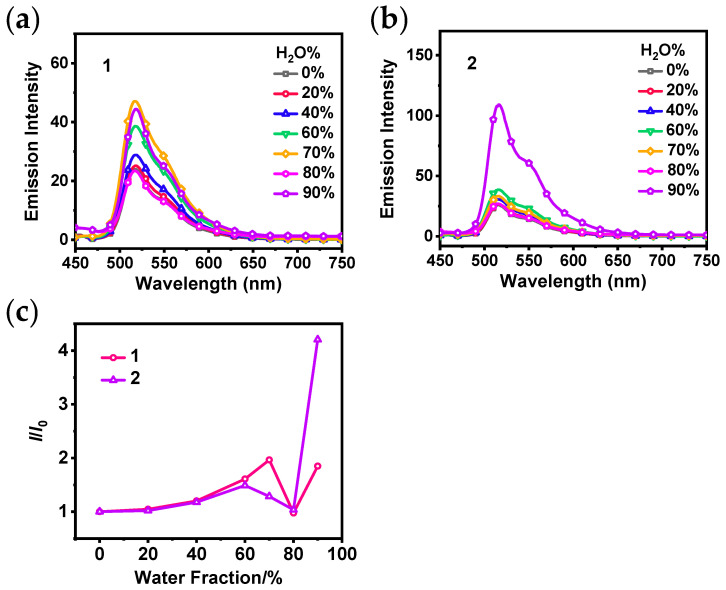
Emission spectra of **1** (**a**) and **2** (**b**) in THF/H_2_O with various water fractions (0–90%) (*c* = 10 μM, *λ*_ex_ = 400 nm); (**c**) line plots of the ratio of the maximum emission intensity (*I*) of **1** and **2** in THF/H_2_O at various water fractions to the emission intensity of their monomers (*I*_0_).

Due to the large conjugation plane of the carbazolyl group, molecular motion may be greatly restricted when close stacking occurs. As a result, there is a high probability that **1** and **2** will exhibit AIPE property. To confirm this conjecture, suspensions of **1** and **2** in THF/H_2_O with various water fractions were prepared, and their emission spectra and UV−Vis absorption spectra were recorded as shown in Figure S5. Figure 2a,b show that the emission intensity of **1** or **2** increases gradually as the water fraction was increased from 0% to 60%. Further increasing the water fraction resulted in the maximum emission intensity of **1** and **2** at 70% and 90%, respectively, indicating the significant AIPE phenomenon. The AIPE phenomenon occurs mainly due to the formation of aggregates. DLS tests of **2** in suspensions at 70%, 80%, and 90% water fractions were conducted to verify the formation of aggregates. The results in Figure S6 exhibit that aggregates are really formed at 70–90% water fractions with small PDIs (<0.1), and the average sizes of the aggregates are 449.3, 389.5, and 190.8 nm, respectively. The ratio plot (Figure 2c) shows that **2** exhibits a higher AIPE property than **1** under the same experimental conditions, which is similar to the findings reported by Yang et al. in 2011 [35]. The introduction of a methyl group into the complex is believed to change the arrangement of **2** in the aggregated state, resulting in tighter intermolecular interactions to restrict intramolecular movement efficiently. This leads to the enhanced luminescence of **2** at high water fractions.

### 3.2. Detection of PA

**Figure 3:**
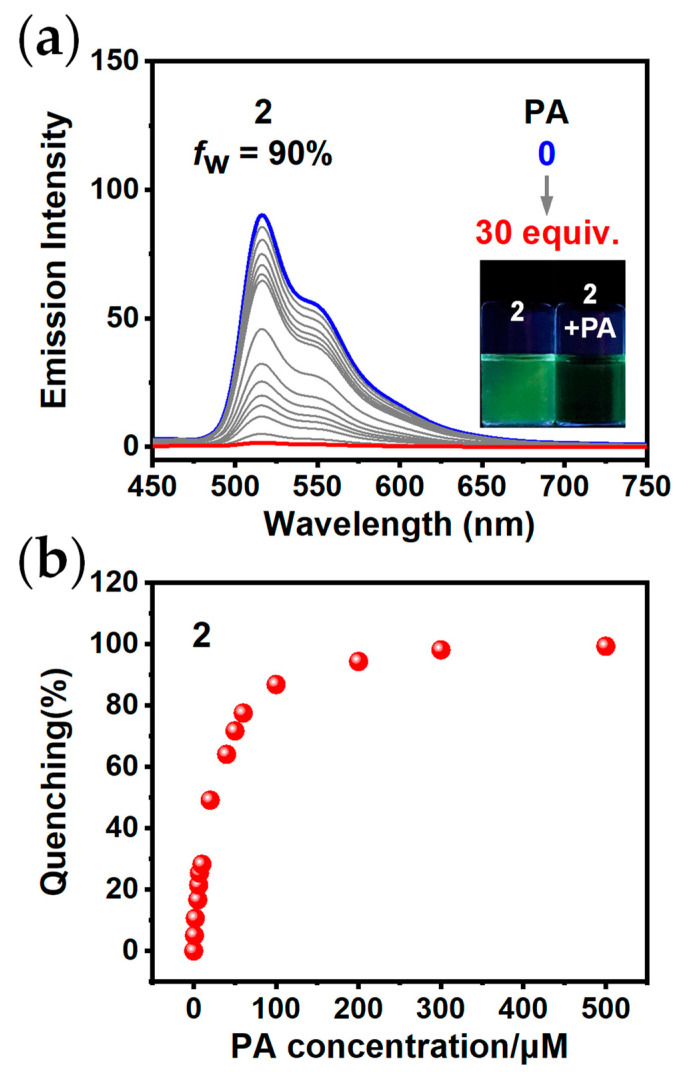
(**a**) Emission spectra of **2** after addition of PA at various concentrations. Insert: The photographs of **2** with PA at a concentration of 0 or 300 μM under 365 nm UV light (*c* = 10 μM, *λ*_ex_ = 400 nm); (**b**) the quenching percentages of **2** after addition of PA at various concentrations.

Notably, **2** exhibits impressive AIPE property in THF/H_2_O, suggesting its potential for the detection of PA in aqueous media. We further conducted the luminescence quenching experiments of **2**. As shown in Figure 3a,b, the emission intensity of **2** decreased progressively as the concentration of PA increased. The quenching efficiency of **2** was 28% when the concentration of PA reached 10 μM. The quenching efficiency of **2** increased significantly to 98% after the addition of PA solution with a concentration of 300 μM (30 equiv.).

**Figure 4:**
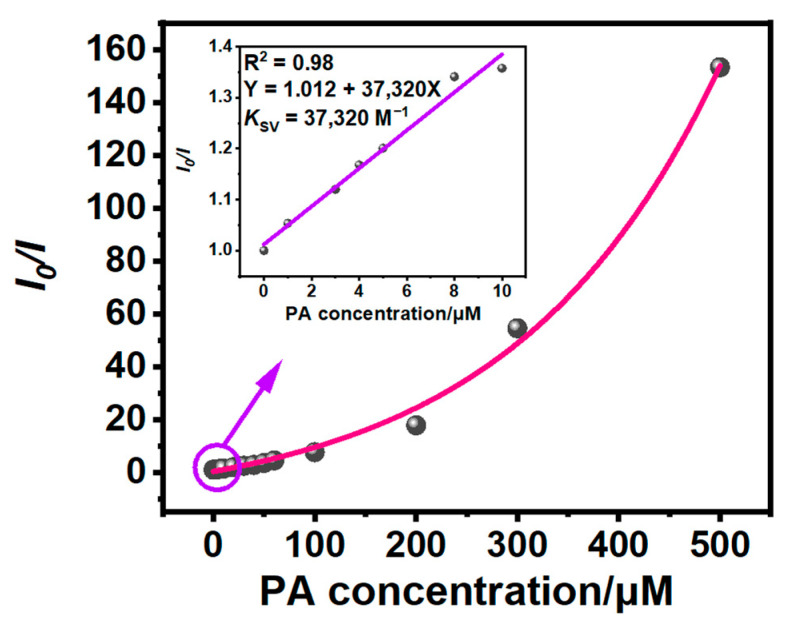
The Stern–Volmer plot of **2** for detecting PA. Insert: The linear part of Stern–Volmer plot in the concentration of PA ranging from 0 to 10 μM.

The quenching constant (*K*_SV_) is usually used as an index to the sensitivity of a sensor to detect PA. To obtain the *K*_SV_ of **2** for PA, a Stern–Volmer plot of **2** was constructed using the ratio of the luminescence intensity without PA (*I*_0_) to the luminescence intensity with PA (*I*) vs. the concentration of PA (Figure 4). The Stern–Volmer plot displays excellent linearity within the concentration of PA range from 0 to 10 μM while within the concentration of PA range from 0 to 500 μM, there is a non-linear relationship. The sensitivity of **2** for PA is assessed utilizing the Stern–Volmer equation: *I*_0_/*I* = *K*_SV_ [Q] + 1 [36]. In the concentration range from 0 to 10 μM, a linear fit is used to calculate the *K*_SV_ of **2** to be 37,320 M^−1^. This value is higher than those of previously reported neutral Ir(III) complexes with carbazolyl groups for PA detection [15,16]. In addition, the LOD of **2** for PA can be calculated using the formula LOD = 3*σ*/*K*, where *σ* represents the standard deviation of 11 blank samples of **2** (Table 1), and *K* represents the slope of the linear relationship between the luminescence intensity *I* and PA concentration [37].

**Figure 5:**
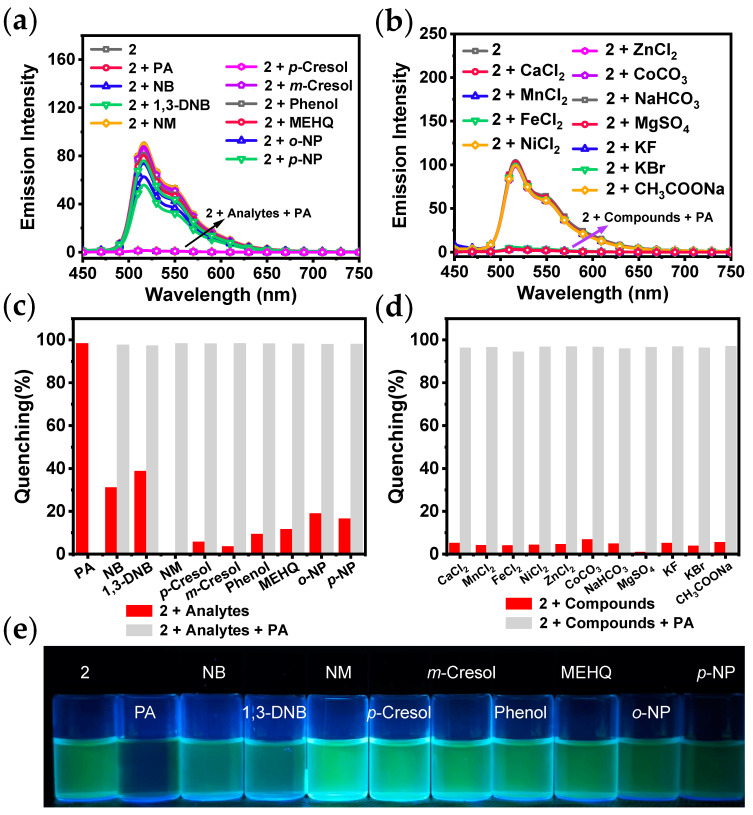
Emission spectra of **2** in the presence of various analytes (**a**), ionic compounds (**b**) (*c* = 10 μM, *λ*_ex_ = 400 nm); quenching percentages of **2** with various analytes (**c**), ionic compounds (**d**) before (red) and after (gray) adding PA; (**e**) photos of the mixtures of **2** in THF/H_2_O with various analytes under 365 nm UV light (*c* = 10 μM).

Selectivity is deemed a key factor influencing the probe performance. Therefore, the luminescence response studies on several common analytes were conducted, including NB, 1,3-DNB, NM, *p*-cresol, *m*-cresol, phenol, MEHQ, *o*-NP, and *p*-NP. The emission spectra can be seen in Figure 5a. Figure 5c shows that the luminescence quenching efficiency of **2** is higher than those of the other analytes after adding PA, demonstrating good selectivity.

In order to further explore selectivity and the anti-interference capacity of **2** and to expand the scope of its application for the detection of PA, various ionic compounds were used for the emission spectroscopic studies, including CaCl_2_, MnCl_2_, FeCl_2_, NiCl_2_, ZnCl_2_, CoCO_3_, NaHCO_3_, MgSO_4_, CuSO_4_, KF, KBr, and CH_3_COONa. Adding solutions of various compounds did not lead to a noticeable alteration in the emission spectra or emission intensity of **2** (Figure 5b). This suggests that the addition of ions has a small impact on the luminescent property of **2** in THF/H_2_O.

Competition experiments were carried out by introducing 30 equiv. of PA solution into suspensions containing various analytes or ionic compounds. As shown in Figure 5c,d, the luminescence quenching efficiency of **2** by PA was found to be almost unaffected in the presence of various competing compounds. Therefore, **2** shows outstanding anti-interference capacity when used to detect PA.

**Figure 6:**
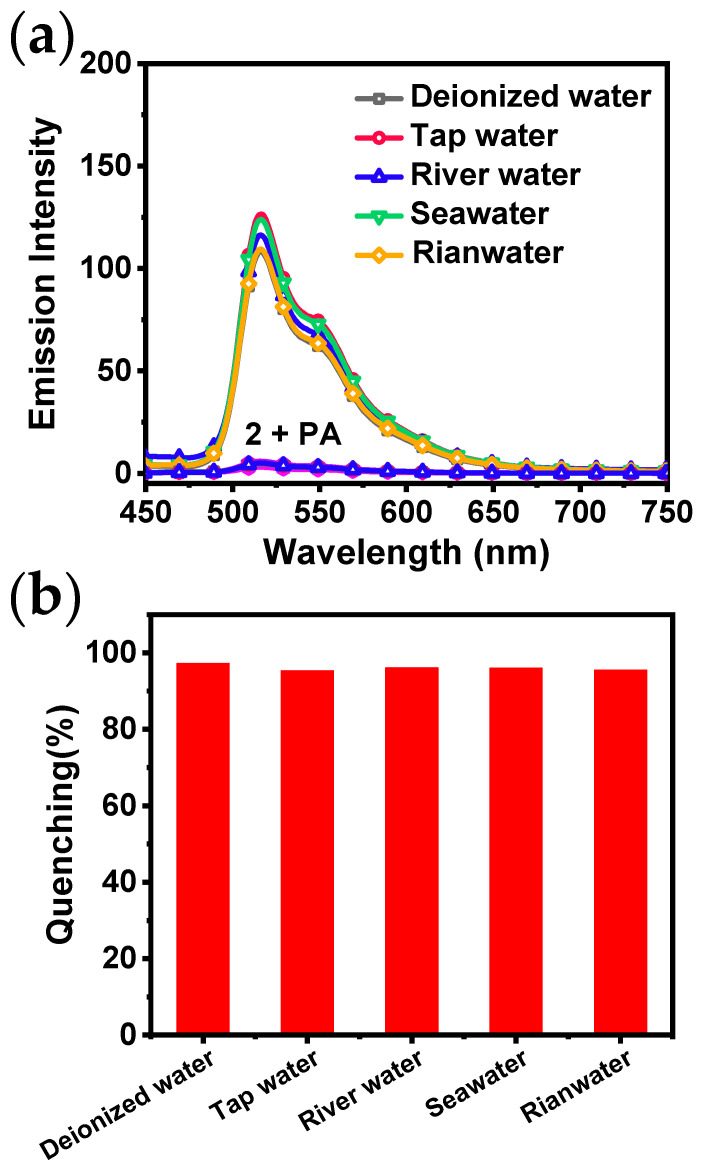
(**a**) Emission spectra of **2** in THF/H_2_O using common water samples (*c* = 10 μM, *λ*_ex_ = 400 nm) with or without PA; (**b**) quenching percentages of **2** towards PA using common water samples.

Water resources represent a crucial component of the ecological environment and are utilized in a multitude of social production and life processes. PA is readily soluble in hot water and has the potential to pollute water sources. Consequently, it is of paramount importance to detect the presence of PA in common water samples. The emission spectra of **2** before and after the addition of PA in various water samples are shown in Figure 6a. In comparison to the luminescence observed within deionized water, the emission spectra of **2** in four common water samples exhibit similar shapes, yet distinct emission intensity. The quenching efficiency of **2** for detecting PA in different water samples is shown in Figure 6b, and the results did not differ significantly. The spectral analysis indicates that the efficiency of luminescence quenching of **2** by PA is considerably higher than that of the impurities present in four common water samples.

### 3.3. Sensing Mechanism

**Figure 7:**
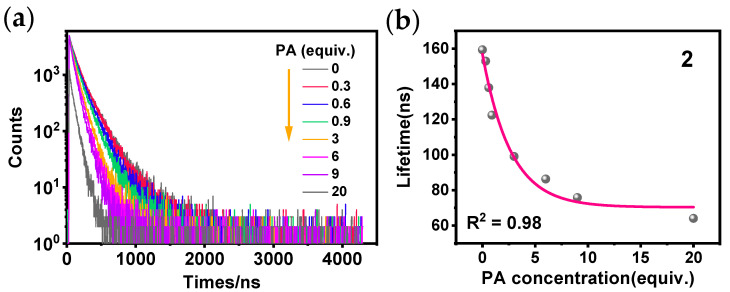
(**a**) Phosphorescence decay traces of **2** in THF/H_2_O after addition of PA at various concentrations; (**b**) phosphorescence lifetime graph of **2** in THF/H_2_O after addition of PA at various concentrations.

Two common quenching mechanisms in fluorescence are static and dynamic quenching [38]. These two quenching mechanisms can be distinguished by changes in the luminescence lifetimes of the sensors [39]. To determine the type of quenching mechanism for compound **2** in the presence of PA, the lifetime decay traces of **2** with the addition of various PA concentrations were recorded (Figure 7a). The lifetimes of **2** in the presence of different concentrations of PA were obtained by fitting with computer software (Fluoracle, version 2.17.2) as shown in Figure 7b. The results indicate that the quenching of **2** is a dynamic quenching process at both low and high concentrations of PA.

**Figure 8:**
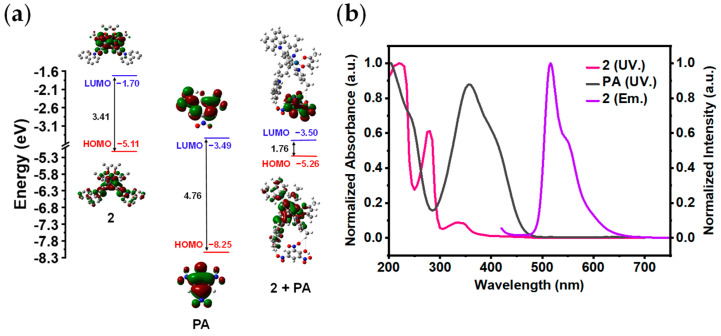
(**a**) Calculated energy level diagram of **2**, PA, and adduct (**2** + PA); (**b**) the normalized UV−Vis absorption spectra of **2** (pink) and PA (black) and normalized emission spectrum of **2** (purple) (*c* = 10 μM, *λ*_ex_ = 400 nm).

In order to investigate what kind of energy transfer or charge transfer actually occurs during the dynamic quenching process, we calculated the LUMOs and highest occupied molecular orbitals (HOMOs) of **2**, adduct (**2** + PA), and PA. Figure 8a shows that the LUMO energy of PA is −3.49 eV, which is lower than that of **2**. This implies that **2** and PA are capable of undergoing PET. The excited-state electrons in the LUMO of **2** are easily transferred to the LUMO of PA rather than returning directly to its own HOMO, resulting in the quenching of the luminescence of **2**. The energy gap of **2** is higher than that of adduct (**2** + PA), indicating the adduct is much more stable. Förster Resonance Energy Transfer (FRET) does not occur during sensing because there is no overlap between the emission spectrum of **2** and the absorption spectrum of PA (Figure 8b). Additionally, the normalized emission spectra of **2** remain almost unchanged after the addition of PA at different concentrations (Figure S11), proving that no other emitting species are formed during the quenching process [15]. Thus, the sensing mechanism of **2** for PA is proposed to be PET.

## 4. Conclusions

In summary, two carbazolyl-modified neutral Ir(III) complexes **1** and **2** were synthesized and fully characterized. Compared to **1**, **2** with a methyl group at the 5-position of the pyridyl ring exhibits a much stronger AIPE property in THF/H_2_O. Additionally, **2** exhibits excellent sensitivity and selectivity for detecting PA in aqueous media, with the *K*_SV_ of 37,320 M^−1^ and an LOD of 0.15 μM. Furthermore, **2** shows high quenching efficiency for PA in various common water samples. Spectroscopic studies and DFT calculations indicate that the sensing mechanism is PET. This work offers renewed insights for the rational structural design of neutral Ir(III) complexes for detecting PA in aqueous media.

## Figures and Tables

**Table 1:** The emission intensity of **2** at 516 nm in 11 blank samples in THF/H_2_O (*f*_w_ = 90%, 10 μM).

Complex 2	Intensity
X1	101.59
X2	101.52
X3	101.33
X4	101.37
X5	101.33
X6	101.22
X7	101.20
X8	101.39
X9	101.21
X10	101.49
X11	101.28
X	101.36
*σ*	0.125

Xi (i = 1, 2, 3…11) represents the emission intensity of each blank sample, X represents the mean value of the emission intensity, i represents the number of blank samples, and the formula for *σ* is described in the Supplementary Materials.

## Data Availability

Data are contained within the article.

## Supplementary Materials

The following supporting information can be downloaded at: https://www.mdpi.com/article/10.3390/s24134074/s1, Figure S1: Synthesis routes of the cyclometalating ligands **L1** and **L2**; Figure S2: Synthetic routes for complexes **1** and **2**; Figure S3: UV−Vis absorption spectra of **1** and **2** in (a) THF and (b) CH_2_Cl_2_; (c) normalized emission spectra of **1** and **2** in THF (10 μM); Figure S4: Phosphorescence decay traces of (a) **1** and (b) **2** in deoxygenated CH_2_Cl_2_ (10 μM); Figure S5: UV−Vis absorption spectra of **1** and **2** at 10 μM in THF/H_2_O with various water fractions; Figure S6: DLS analysis of **2** at (a) 70%, (b) 80%, and (c) 90% water fractions (10 μM, THF/H_2_O); Figure S7: Calculated energy level diagrams of **1** and **2**; Figure S8: The emission spectra of **2** in 11 blank samples in THF/H_2_O (*f*_w_ = 90%, 10 μM); Figure S9: The linear graph of the emission intensity of **2** vs. the concentrations of PA; Figure S10: Photos of the mixtures of **2** in THF/H_2_O with various ionic compounds under 365 nm UV light; Figure S11: The normalized emission spectra of **2** in THF/H_2_O after addition of different concentrations of PA; Figure S12: ^1^H NMR spectrum of **1** in CDCl_3_; Figure S13: ^13^C NMR spectrum of **1** in CDCl_3_; Figure S14: ^1^H NMR spectrum of **2** in CDCl_3_; Figure S15: ^13^C NMR spectrum of **2** in CDCl_3_; Figure S16: The HRMS of **1**; Figure S17: The HRMS of **2**; Table S1: Photophysical data of complexes **1** and **2**. Reference [27] are cited in the Supplementary Materials.
