# Supplementary material for: Carbazolyl-Modified Neutral Ir(III) Complexes for Efficient Detection of Picric Acid in Aqueous Media
Source: Sensors (Basel). 2024 Jun 22;24(13):4074. doi: 10.3390/s24134074 (PMC11244125; doi:10.3390/s24134074)
Supplement: Supplementary file 1 [file sensors-24-04074-s001.zip › sensors-3040824-supplementary.pdf]

Supplementary data for

Carbazolyl-Modified Neutral Ir(III) Complexes for Efficient Detection of Picric Acid in Aqueous Media

Jiangchao Xu, Liyan Zhang, Yusheng Shi * and Chun Liu *

State Key Laboratory of Fine Chemicals, Frontier Science Center for Smart Materials, School of Chemical Engineering, Dalian University of Technology, Linggong Road 2, Dalian 116024, China. E-mail: cliu@dlut.edu.cn

Contents

Synthesis and characterization *S2-S3*

Photophysical and AIPE properties of **1** and **2** *S4-S6*

Theoretical calculations *S7*

Sensing of PA *S8-S9*

NMR spectra and HRMS of **1** and **2** *S10-S12*

References *S13*

Synthesis and characterization

Synthesis of ligands L1 and L2

Cyclometalating ligands were synthesized with reference to the methods reported in the literature [1]. Brominated heterocyclic aromatic hydrocarbons (0.5 mmol), 4-(9-carbazolyl)phenylboronic acid (0.75 mmol, 215.35 mg), K_2CO_3 (1 mmol, 138.21 mg), and $Pd(OAc)_2$ (1.5 mol%, 1.69 mg) were added to a mixture of 8 mL of anhydrous $EtOH/H_2O$ (3:1, V/V), and the reaction was carried out under air at 80°C for 10–30 min. After the reaction, the cyclometalating ligands **L1** and **L2** were further purified by silica column chromatography using petroleum ether and ethyl acetate as eluents.

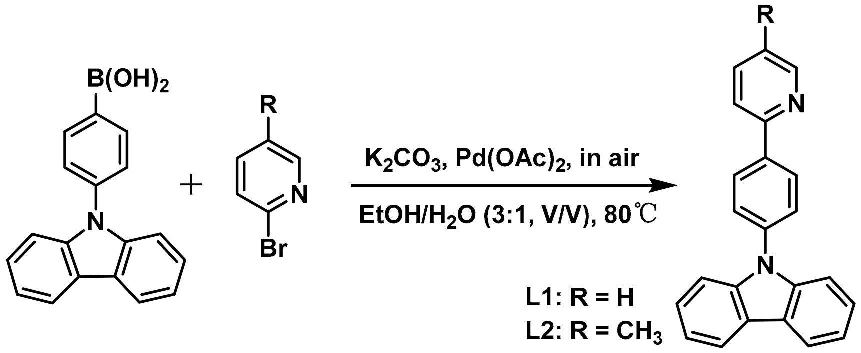

Figure S1 Synthesis routes of the cyclometalating ligands **L1** and **L2**.

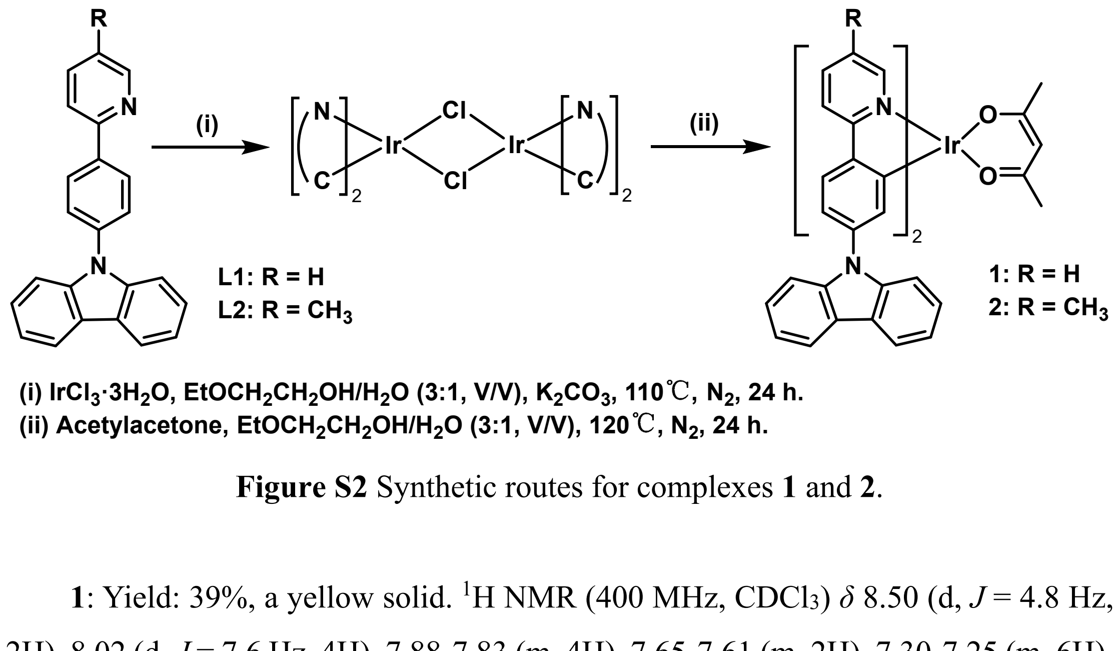

Figure S2 Synthetic routes for complexes **1** and **2**.

1: Yield: 39%, a yellow solid. ^{1}H NMR (400 MHz, $CDCl_3$) δ 8.50 (d, J = 4.8 Hz, 2H), 8.02 (d, J = 7.6 Hz, 4H), 7.88-7.83 (m, 4H), 7.65-7.61 (m, 2H), 7.30-7.25 (m, 6H), 7.20-7.17 (m, 6H), 7.15-7.12 (m, 2H), 7.06 (d, J = 6.8 Hz, 2H), 6.46 (d, J =2.0 Hz, 2H), 5,34 (s, 1H), 1.88 (s, 6H). ^{13}C NMR (100 MHz, $CDCl_3$) δ 184.92, 167.63, 148.63, 148.26, 143.72, 140.36, 137.57, 137.37, 130.78, 125.51, 124.80, 123.19, 121.71, 119.91,

119.51, 118.70, 118.67, 110.51, 100.79, 28.88. MALDI-TOF-MS (*m*/*z*) calcd. for $C_{51}H_{37}N_4O_2Ir$ $[M]^+$: 930.2546, found: 930.2491.

2: Yield: 45%, a yellow solid. ^1H NMR (400 MHz, $CDCl_3$) δ 8.31 (d, J = 5.6 Hz, 2H), 8.02 (d, J = 7.6 Hz, 4H), 7.80 (d, J = 8.0 Hz, 2H), 7.66 (s, 2H), 7.29-7.25 (m, 6H), 7.19-7.16 (m, 6H), 7.12-7.09 (m, 2H), 6.88 (d, J = 6.0 Hz, 2H), 6.46 (d, J = 2.0 Hz, 2H), 5.31 (s, 1H), 2.45 (s, 6H), 1.87 (s, 6H). ^{13}C NMR (100 MHz, $CDCl_3$) δ 184.80, 164.91, 148.02, 147.80, 143.99, 140.38, 138.17, 137.03, 131.54, 130.77, 125.44, 124.22, 123.12, 119.86, 119.42, 118.53, 118.24, 110.54, 100.79, 28.96, 18.51. MALDI-TOF-MS (*m*/*z*) calcd. for $C_{53}H_{41}N_4O_2Ir$ $[M]^+$: 958.2859, found: 958.2875.

Photophysical and AIPE properties

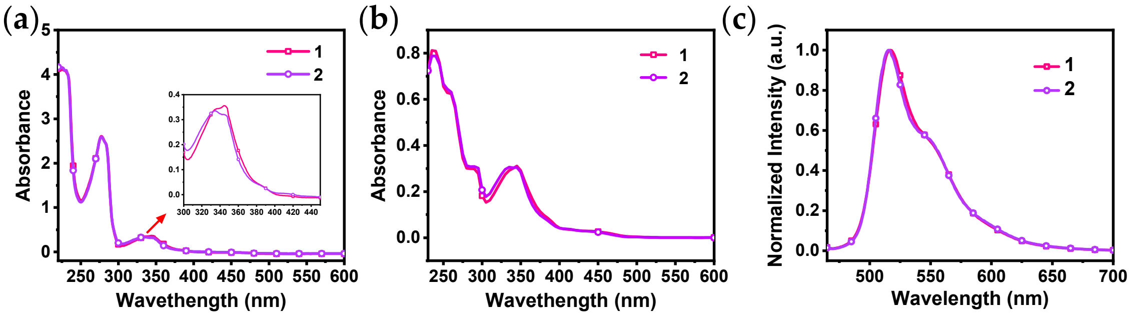

Figure S3 UV–Vis absorption spectra of **1** and **2** in (a) THF and (b) CH_2Cl_2; (c) normalized emission spectra of **1** and **2** in THF (10 μM).

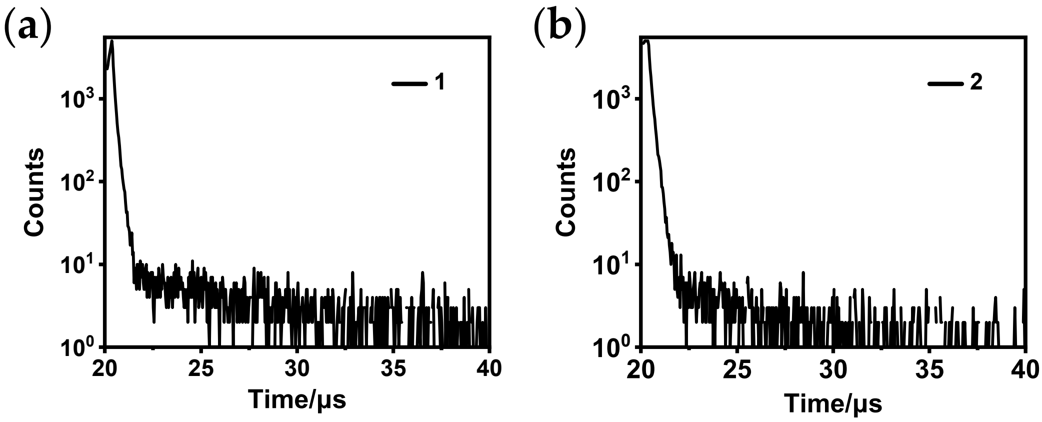

Figure S4 Phosphorescence decay traces of (a) **1** and (b) **2** in deoxygenated CH_2Cl_2 (10 μM).

Table S1 Photophysical data of complexes **1** and **2**.

Complexes	λ_{abs} [a] (nm)	λ_{em} [b] (nm)	Φ_{PL} [c]	τ [d] (μs)
**1**	**278** (2.60)<br>344 (0.36)	**517**	0.18	1.92
**2**	**278** (2.59)<br>334 (0.34)	**516**	0.27	1.77

[a] Measured in THF at a concentration of 10 μM; [b] the maximum emission values are bold; [c] the quantum yields ($\Phi_{solution}$) in deoxygenated CH_2Cl_2 were measured with [Ir(ppy)$_2$(acac)] (Φ_{PL} = 0.34) as a standard; [d] measured in deoxygenated CH_2Cl_2.

Figure S5 shows the UV–Vis absorption spectra of **1** and **2** in THF/H_2O with various water fractions (c = 10 μM). The spectra indicate a peak at 278 nm for **1** and **2** in the range of 0–60% water fraction. As the water fraction increases, the absorbance gradually decreases, but the absorption wavelength remains constant. The absorption spectra do not show significant changes beyond 300 nm. At the 70% water fraction, the absorbances of **1** and **2** decrease continuously at 278 nm, and there are no significant changes in the absorption spectra of **1** and **2** beyond 300 nm. However, at 80% and 90% water fractions, the absorption spectra of **1** and **2** change significantly, resulting in a red shift at different degrees and increased absorbances beyond 300 nm. It is worth noting that the absorption spectra of **1** and **2** change significantly only at 80% and 90% water fractions. Changes in the absorption spectra can be attributed to the formation of aggregates at high water fractions.

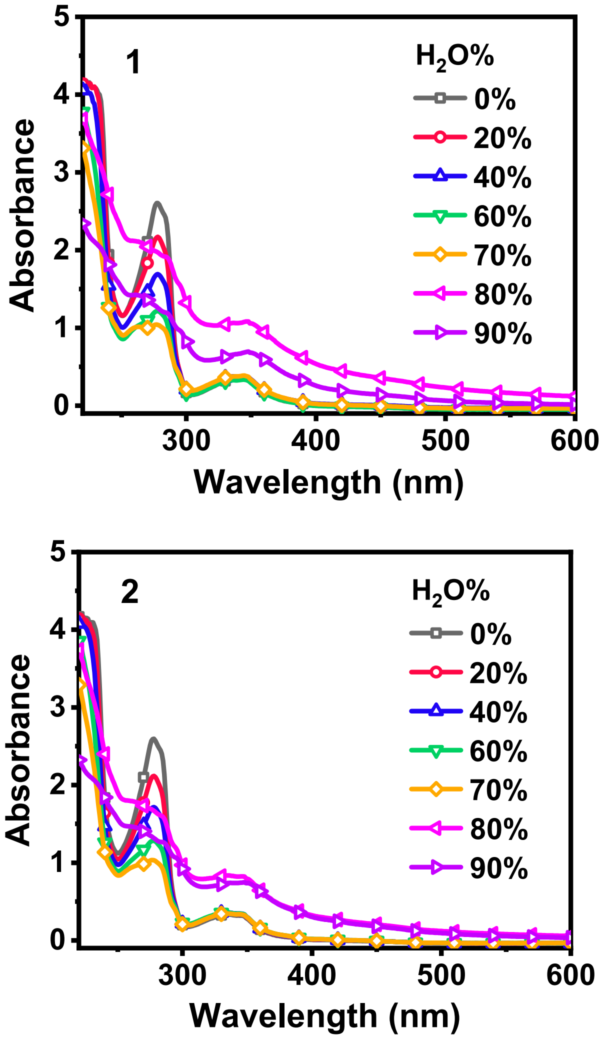

Figure S5 UV–Vis absorption spectra of **1** and **2** at 10 μM in THF/H_2O with various water fractions.

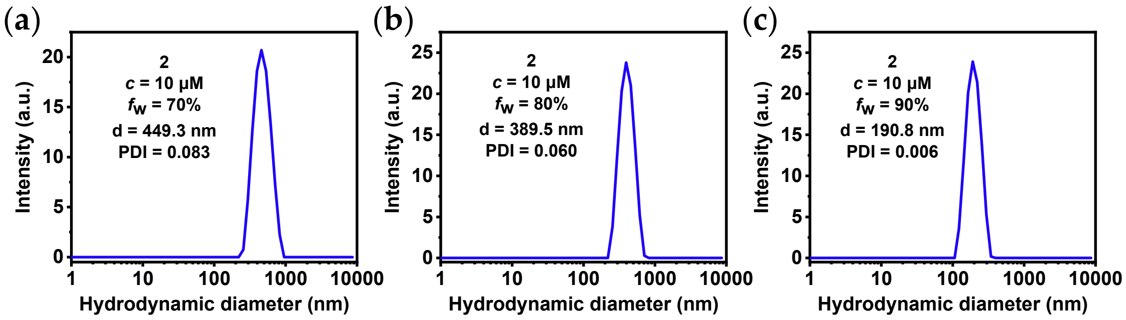

Figure S6 DLS analysis of **2** at (a) 70%, (b) 80%, and (c) 90% water fractions (10 μM, THF/H_2O).

Theoretical calculations

DFT calculations were employed to analyze the electronic distributions of **1** and **2**. The highest occupied molecular orbitals (HOMOs) of both complexes are primarily located on the iridium centers and the phenyl and carbazolyl moieties of the cyclometalating ligands, whereas the lowest unoccupied molecular orbitals (LUMOs) are primarily distributed on the aryl and pyridine rings of the cyclometalating ligands. As shown in **Figure S7**, the energy gaps (E_g) of **1** and **2** are 3.43 and 3.41 eV, respectively. It is evident that introducing a methyl group results in a small elevation of the HOMO and LUMO levels of the complexes.

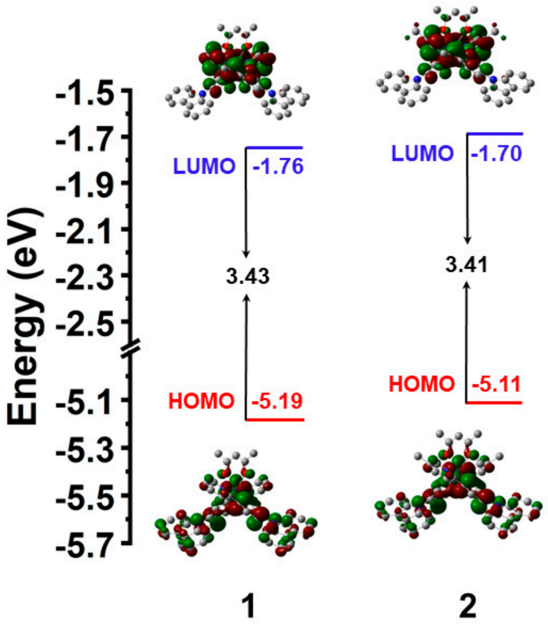

Figure S7 Calculated energy level diagrams of **1** and **2**.

Sensing of PA

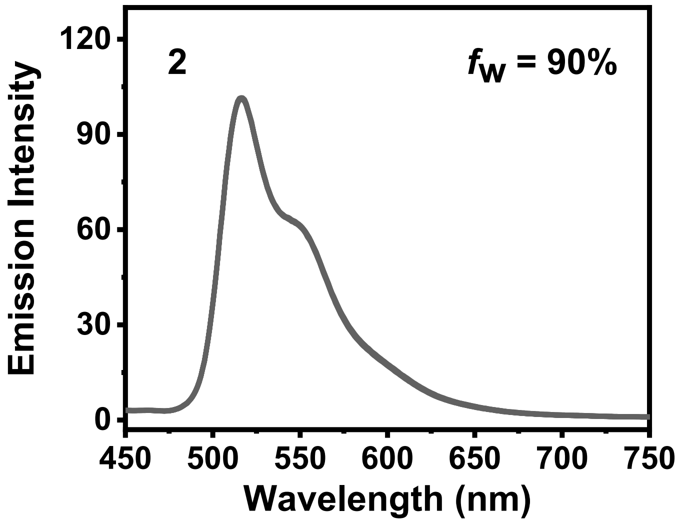

Figure S8 The emission spectra of **2** in 11 blank samples in THF/H_2O (f_w = 90%, 10 μM).

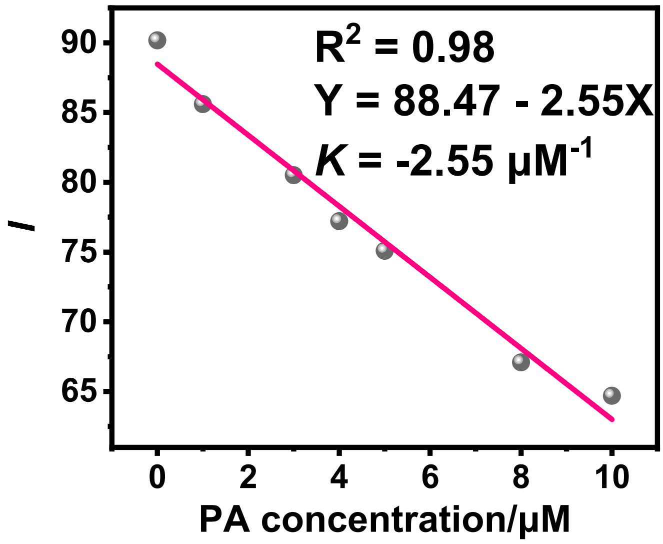

Figure S9 The linear graph of the emission intensity of **2** vs. the concentrations of PA.

The value of σ for **2** was calculated according to the following equation:

$$\sigma = \sqrt{\frac{\sum(X_i - X)^2}{n-1}}$$

X_i (i = 1, 2, 3…11) represents the emission intensity of each blank sample, X represents the mean value of the emission intensity, and n represents the number of blank samples.

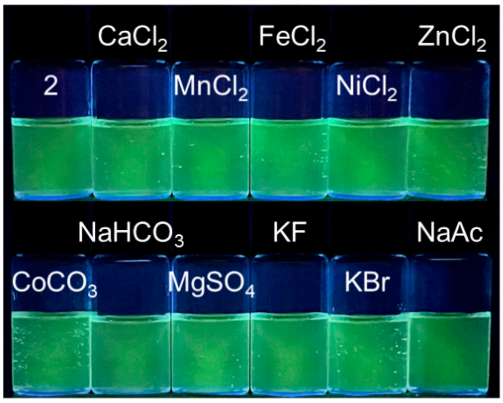

Figure S10 Photos of the mixtures of **2** in THF/H_2O with various ionic compounds under 365 nm UV light.

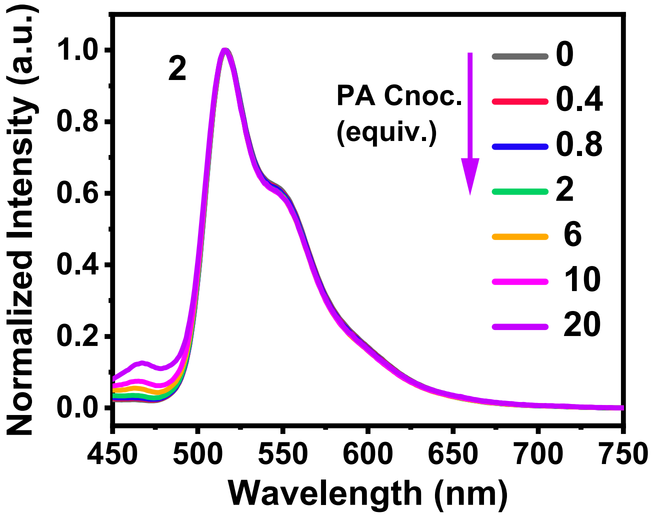

Figure S11 The normalized emission spectra of **2** in THF/H_2O after addition of different concentrations of PA.

NMR spectra and HRMS of 1 and 2

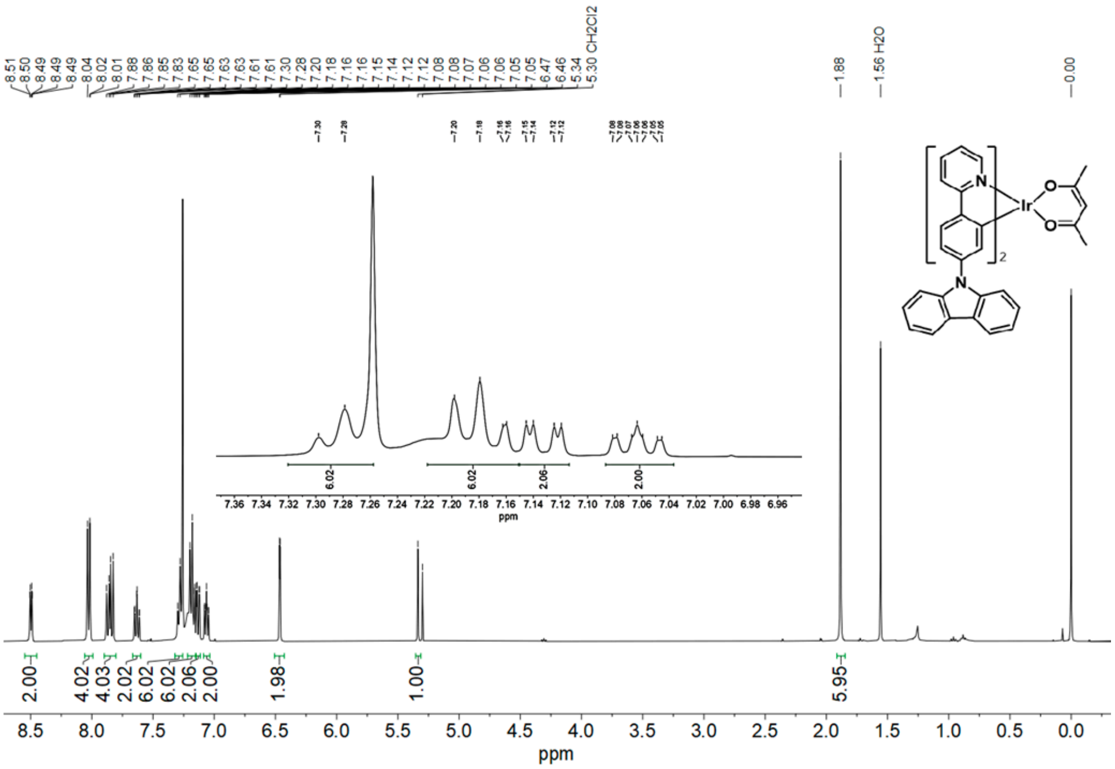

Figure S12 ^{1}H NMR spectrum of **1** in $CDCl_3$.

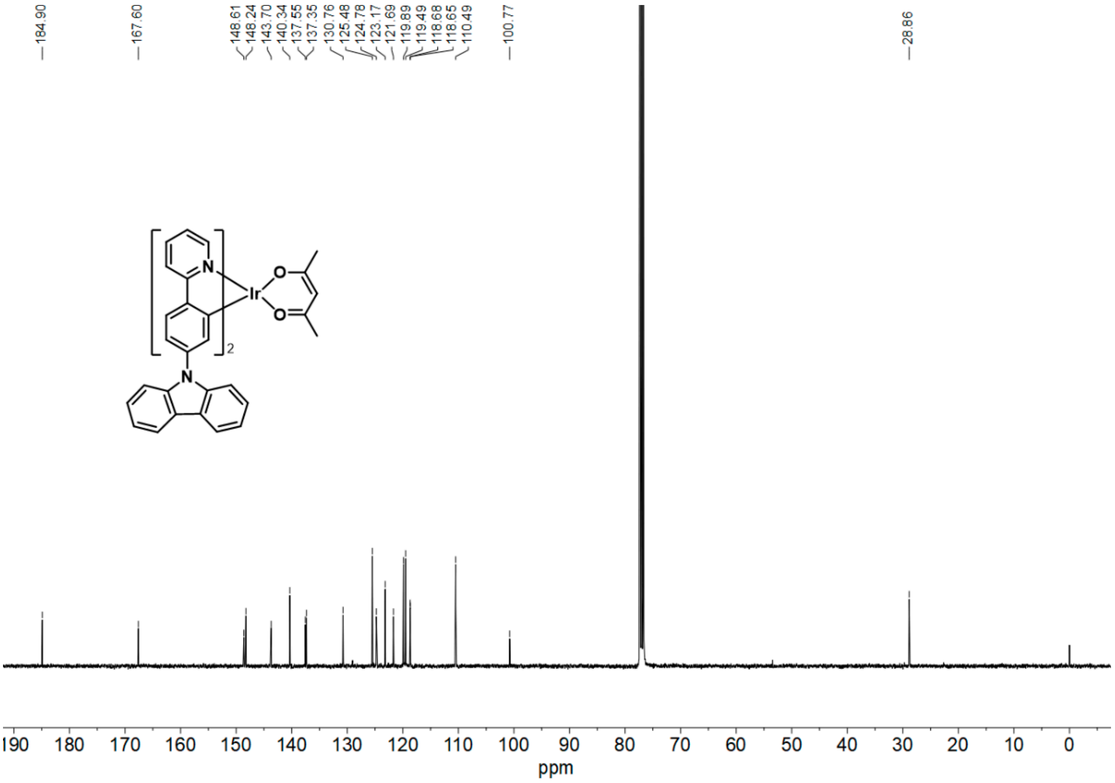

Figure S13 ^{13}C NMR spectrum of **1** in $CDCl_3$.

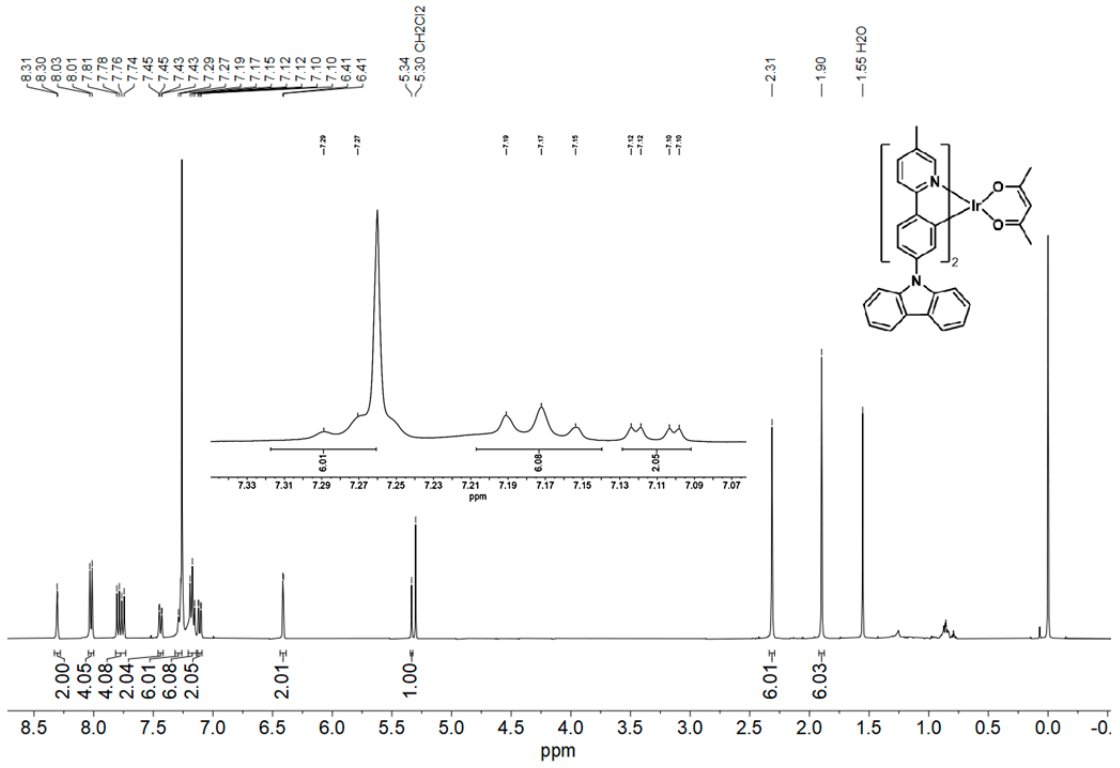

Figure S14 ^{1}H NMR spectrum of **2** in $CDCl_3$.

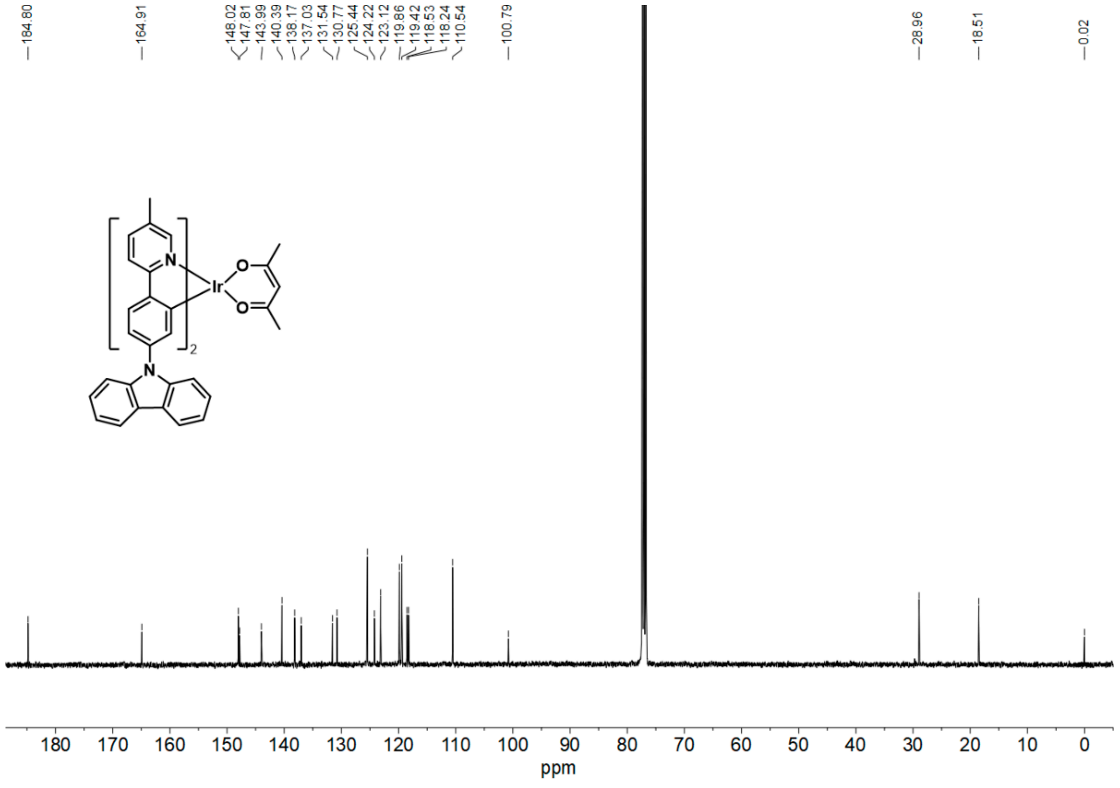

Figure S15 ^{13}C NMR spectrum of **2** in $CDCl_3$.

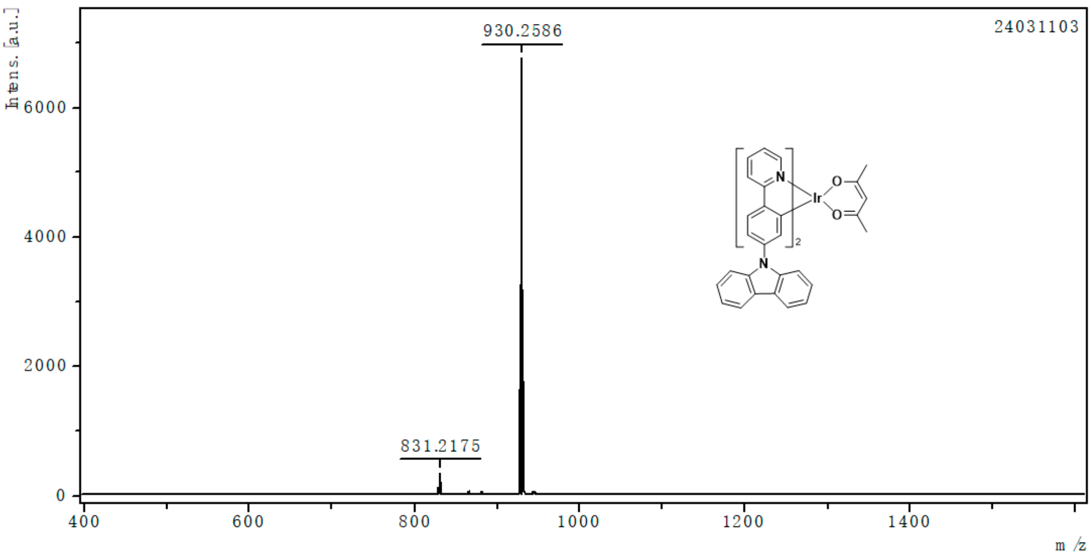

Figure S16 The HRMS of **1**.

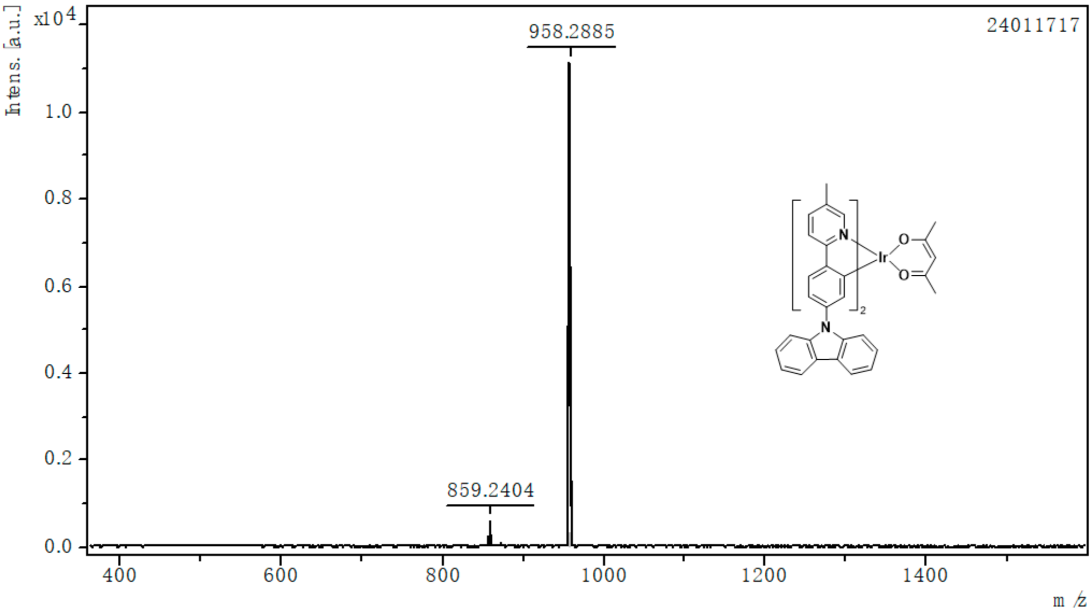

Figure S17 The HRMS of **2**.

Reference

[1] Liu, C.; Rao, X.; Lv, X.; Qiu, J.; Jin, Z. Substituent effects on the photophysical and electrochemical properties of iridium(III) complexes containing an arylcarbazolyl moiety. *Dyes Pigm.* **2014**, *109*, 13-20.
